# Effects of 21 days of bed rest and whey protein supplementation on plantar flexor muscle fatigue resistance during repeated shortening contractions

**DOI:** 10.1007/s00421-020-04333-5

**Published:** 2020-03-04

**Authors:** Alessandra Bosutti, Edwin Mulder, Jochen Zange, Judith Bühlmeier, Bergita Ganse, Hans Degens

**Affiliations:** 1 Department of Life Sciences, and Centre for Neuroscience B.R.A.I.N, University of Trieste, Via A. Fleming 22, 34127 Trieste, Italy; 2 Institute of Aerospace Medicine, German Aerospace Center DLR, Cologne, Germany; 3 Department of Child and Adolescent Psychiatry, University Hospital Essen, University of Duisburg-Essen, Essen, Germany; 4 Department of Life Sciences, Musculoskeletal Science and Sports Medicine Research Centre, Manchester Metropolitan University, Manchester, UK; 5 Institute of Sport Science and Innovations, Lithuanian Sports University, Kaunas, Lithuania; 6 University of Medicine and Pharmacy of Targu Mures, Târgu Mureș, Rumania

**Keywords:** Spaceflight, Fluid shift, Bed rest, Shortening contraction, Muscle fatigue, Plantar flexors, Pi, ATP, ^31^P nuclear magnetic resonance spectroscopy, Muscle oxygenation

## Abstract

**Purpose:**

Space flight and bed rest (BR) lead to a rapid decline in exercise capacity. Whey protein plus potassium bicarbonate diet-supplementation (NUTR) could attenuate this effect by improving oxidative metabolism. We evaluated the impact of 21-day BR and NUTR on fatigue resistance of plantar flexor muscles (PF) during repeated shortening contractions, and whether any change was related to altered energy metabolism and muscle oxygenation.

**Methods:**

Ten healthy men received a standardized isocaloric diet with (*n* = 5) or without (*n* = 5) NUTR. Eight bouts of 24 concentric plantar flexions (30 s each bout) with 20 s rest between bouts were employed. PF muscle size was assessed by means of peripheral quantitative computed tomography. PF muscle volume was assessed with magnetic resonance imaging. PF muscle force, contraction velocity, power and surface electromyogram signals were recorded during each contraction, as well as energy metabolism (^31^P nuclear magnetic resonance spectroscopy) and oxygenation (near-infrared spectroscopy). Cardiopulmonary parameters were measured during an incremental cycle exercise test.

**Results:**

BR caused 10–15% loss of PF volume that was partly recovered 3 days after re-ambulation, as a consequence of fluid redistribution. Unexpectedly, PF fatigue resistance was not affected by BR or NUTR. BR induced a shift in muscle metabolism toward glycolysis and some signs of impaired muscle oxygen extraction. NUTR did not attenuate the BR-induced-shift in energy metabolism.

**Conclusions:**

Twenty-one days’ BR did not impair PF fatigue resistance, but the shift to glycolytic metabolism and indications of impaired oxygen extraction may be early signs of developing reduced muscle fatigue resistance.

**Electronic supplementary material:**

The online version of this article (10.1007/s00421-020-04333-5) contains supplementary material, which is available to authorized users.

## Introduction

Exposure to real or simulated microgravity like head-down tilt bed rest leads to loss of muscle mass and a rapid decline in exercise capacity (Zange et al. 1997; Trappe et al. 2007; LeBlanc et al. 1988; Korya et al. 2018). Similar changes are often observed in patients during prolonged periods of immobilisation (Trethewey et al. 2019). The severity of muscle atrophy increases with the duration of spaceflight or immobilisation and is, despite available countermeasures, a major concern for long-term space missions (Fitts et al. 2001; Rittweger et al. 2018).

The loss of muscle mass is primarily attributable to the decrease in muscle loading and is accompanied by a reversible rapid decline in muscle force and power generating capacity, and increased susceptibility to contraction-induced muscle damage upon return to Earth (Rittweger et al. 2018; Narici and de Boer 2011).

In addition to the loss of muscle force and power generating capacity, the decrease in weight-bearing activity may also result in a reduced muscle fatigue resistance, where fatigue is defined as a reversible reduction in force or power generating capacity during repeated or prolonged sustained contractions (Enoka and Stuart, 1992; Zange et al. 1997; Korya et al. 2018; Berg et al. 1993; Mulder et al. 2007). This latter response to disuse is, however, equivocal where some studies have reported an unaltered (Semmler et al. 2000; Korya et al. 2018; Duchateau 1995; Witzman et al. 1983; Clark 2009; Weber et al. 2014) or even an increased endurance capacity (Semmler et al. 2000; Vanderborne et al. 1998), either following spaceflight, bed rest or other models of disuse. Such inconsistencies could originate from the fact that the effects of microgravity on neuromuscular performance are dependent on the type of exercise task and employed muscle groups (Clark et al. 2007), and the duration of disuse. Moreover, a large proportion of the current microgravity/bed rest studies employ maximal isometric exercise, whereas in daily life most contractions are shortening, and consequently power generating contractions. Therefore, it remains to be seen how bed rest or spaceflight affects muscle fatigue resistance during shortening contractions that resemble contractions encountered during daily life. If microgravity is associated with a reduction in muscle fatigue resistance, in particular during shortening contractions, then this represents an additional constraint on the work performance and safety of astronauts in space.

The fatigue resistance of a single muscle fibre or motor unit is positively related to its oxidative capacity (Degens and Veerkamp 1994), and oxidative muscles have a higher fatigue resistance than glycolytic muscles. Recently, we found that 19 days’ bed rest resulted in a significant reduction in the oxidative capacity of muscle fibres in the soleus and vastus lateralis muscles in the absence of significant muscle fibre atrophy that was accompanied by a reduction in the whole-body maximal oxygen uptake (V′O_2max_) (Bosutti et al. 2016). Such a reduction in the ability to aerobically generate ATP may cause decrements in phosphocreatine (PCr) re-synthesis (Kappenstein et al. 2014; Kemp et al. 1993), something indeed shown in the SARCOLAB pilot study (Rittweger et al. 2018). An accelerated rise in inorganic phosphate (P_i_) and intracellular acidification are all factors that impair cross-bridge cycling and are associated with the development of muscle fatigue (Fitts 2008; Fitts et al. 2001; Jones, 2010). In other words, the low oxidative capacity we observed previously after 19 days of bed rest (Bosutti et al. 2016) may be associated with impaired energy metabolism and an earlier onset of fatigue during repeated shortening contractions.

Changes in cardiac and vascular functions caused by prolonged disuse may also contribute to lower maximal oxygen uptake and fatigue resistance. Indeed, it has been suggested that an initial decrease in convective O_2_ transport is, with longer exposure to microgravity, followed by a decrease in diffusive O_2_ transport (Ade et al. 2015). Such changes should be reflected by an impaired muscle oxygenation and an earlier onset of increased fatigue in working muscles (Amann and Calbet 2008). In addition, reductions in muscle fluid content could affect muscle endurance and strength, either by reducing the actin myofilament spacing, that may result in a slowing of the muscle and consequently reduce power, or by inducing a loss of mitochondria, with potential impact on muscle energy metabolism (Degens and Wüst 2018).

There is a large interest in designing countermeasures to prevent or mitigate microgravity-induced muscle wasting and dysfunction (Pavy-Le Traon et al. 2007; Rittweger et al. 2015). An integrated intervention consisting of amino acid/protein supplementation and exercise has been recommended to sustain muscle mass during prolonged bed rest (Blottner et al. 2014; English and Paddon-Jones 2010) and recently we showed that a WP plus potassium bicarbonate (KHCO_3_) supplement attenuated the reduction in muscle oxidative capacity following 19 days of bed rest (Bosutti et al. 2016). Given the importance of aerobic metabolism in fatigue resistance, it remains to be seen whether WP + KHCO_3_ will attenuate a bed rest-induced reduction in muscle fatigue resistance.

The main aim of the present study was, therefore, to assess to what extent 21 days of bed rest with or without whey supplement affected plantar flexor muscle fatigue resistance during repeated shortening contractions, and whether any changes were related to alterations in energy metabolism. We hypothesised that bed rest results in a faster accumulation of Pi and H^+^, which leads to slowing of dynamic contractions. Furthermore, we analysed whether a decrease in muscle hydration consequent to the fluid shift during head down tilt bed rest was associated with force loss following bed rest.

## Methods

### Study design

The Medium-Term Bed Rest Whey protein (MTBR/MEP) study was conducted at the Institute of Aerospace Medicine of the German Aerospace Centre in Cologne, Germany. The independent ethics committee of the Ärztekammer Nordrhein, Düsseldorf, Germany approved the study protocol (Medium-term whey protein [MEP]—bed rest study, #2,010,426 from 13/05/2011). The study was conducted in accordance with ethical principles stated in the Declaration of Helsinki (version placed at the time of the experiments) and adhered to the European Space Agency bed rest standardization plan (version 1.5). Participants provided informed consent and were allowed to drop out from the study at any moment.

The overall purpose of the study was to assess the mitigating effect of a nutritional countermeasure, consisting of WP and alkaline mineral salt supplementation (KHCO_3_), on muscle atrophy and bone loss during 21 days of 6° head-down tilted bed rest (HDT + 21). Details of the study design have been described elsewhere (Bühlmeier et al. 2014) and are also reported under www.clinicaltrials.gov (Identifier: NCT01655979).

The original study was conducted over two campaigns in a controlled randomized cross-over design. As ^31^P NMR spectra could not be obtained for the *R* + 1 and *R* + 28 sessions of the 2nd campaign as a result of hardware failure of the magnet, we present the functional and bioenergetics datasets obtained from the first campaign only.

At the start of the campaign, 10 healthy men were randomly assigned to start with 21 days of bed rest with either standardized diet (BR) or daily nutritional supplementation (WP + KHCO_3_ intervention, NUTR). The campaign consisted of 7 days of baseline data collection (BDC-7 through BDC-1), 21 days of 6°-HDT bed rest (HDT + 1 through HDT + 21) and 6 days of in-house recovery (*R* + 0 through *R* + 5). Follow-up measurements were also done at 3 (*R* + 3), 14 (*R* + 14) and 28 (*R* + 28) days after completion of bed rest.

### Participants

The participants (healthy, non-smoking men, aged between 23 and 43 years with a body mass index between 20 and 25 kg·m^−2^), were recruited after successfully completing medical and psychological screening. Potential participants with one or more of the following conditions were excluded: drug consumption, alcohol consumption, vegetarianism, migraine, claustrophobia, hiatal hernia, disorders of calcium or bone metabolism, history of orthostatic intolerance or vestibular disorders, muscle/cartilage/joint/bone diseases, chronic back pain, chronic hypertension, intraocular hypertension, iron deficiency, anaemia, hyperhomocysteinaemia, hyper/hypourecaemia, hyper/hypocalcaemia, diabetes, obesity, arthritis, hyperlipidaemia, any infectious or hepatic disease, or a positive result of thrombophilia screening (ATIII, Protein C and S, F-V-Leiden, Prothrombin, Lupus-partial thromboplastin time) (Bühlmeier et al. 2014). Of the ten selected men who had given their written informed consent, one participant dropped out of the study after the first campaign.

### Standardized diet and nutritional intervention

During the study, five participants (BR) received a weight-maintaining diet that was individually tailored and strictly controlled to minimise any impact of differences in nutrient intake on outcome measures. Resting metabolic rate (RMR) was measured during the first day in the ward and energy intake was based on 160% RMR during the adaptation and recovery phases and 120% RMR during bed rest (Bühlmeier et al. 2014). Protein intake was set at 1.2 g·kg body mass^−1^·day^−1^. The intake of all nutrients was defined according to the Dietary Reference Intakes and strictly controlled by tolerance limits for each nutrient. Following physically demanding experiments there was an additional fluid and energy intake in the form of water and diluted apple juice to compensate for sweat and energy loss. Caffeine, alcohol and flavour enhancers were excluded from the diet.

The nutritional intervention (NUTR) was a combined supplementation of 0.6 g whey protein·kg body mass^−1^·day^−1^ (Diaprotein®, Dr. SteudleInc, Krueger GmbH) plus 90 mmol KHCO_3_·day^−1^. The additional protein (total protein intake: 1.8 g protein·kg body mass^−1^·day^−1^) isocalorically replaced fat and carbohydrates in a ratio of 1:1 (Bühlmeier et al. 2014). While the high-protein intake in the NUTR condition led to a potentially moderately acidifying diet (potential renal acid load: 13 ± 1 mEq·day^−1^) the alkaline urine content confirmed an alkali over acid production, suggesting that the 90 mmol KHCO_3_ effectively prevented this.

### Plantar flexor muscle size

The anatomical plantar flexor (PF) muscle cross-sectional area (CSA_PF_) was assessed with pQCT imaging (Stratec XCT 3000, Stratec Medizintechnik GmbH, Pforzheim, Germany) at BDC-6, HDT + 21 and *R* + 28 at 33% tibia length (0% proximal) of the left leg.

An operator blinded to the condition manually outlined the PF muscles and results were averaged. Threshold and filter settings were optimized for muscle analysis (Stratec, XTC 3000 software version 6.20).

The volumes of the *m. soleus* (SOL), *m. gastrocnemius lateralis *(GL) and * m. gastrocnemius medialis* (GM) were further determined by MRI at 1.5 T using a Siemens Symphony Tim scanner. Therefore, a series of ≥ 62 axial images from the lower leg were captured before bed rest (BL), after 20 days’ bed rest (HDT + 20) and at 3 days after bed rest (*R* + 3). A flash sequence was used with 10° flip angle, repetition time 6.82 ms, echo time 2.38 ms and a pixel matrix of 256 × 192 pixels at 1.05 mm × 1.05 mm pixel size, 5 mm slice thickness. To ensure matching anatomical positions at each time point, the first MRI slice with the appearance of the *vena saphena parva*, *nervus cutaneous surae medialis*, *vena saphena magna*, or *nervus cutaneous surae lateralis* served as a reference. In all slices the SOL, GL and GM were manually outlined and cross-sectional areas calculated in ImageJ (ImageJ software; https://imagej.nih.gov, U.S.A.). The volume of each muscle was calculated as the sum of the CSA in each slice ‘times’ slice thickness (5 mm).

### T2 as a marker of water shifts and water-binding to protein in muscle

In muscle tissue the time constant of the transverse spin relaxation T2 is predominantly determined by the relative contents of free water versus immobilised water binding at protein or other macromolecules. A decrease in water content without protein loss would be indicated by a decrease in T2, whereas an increase in water at constant protein content would result in an increase in T2 (Patten et al. 2003). Therefore, measurements of T2 in combination with volume measurements by image segmentation provide information about volume changes including protein synthesis or degradation or volume changes predominantly caused by water-shifts. At BL, HDT + 20 and *R* + 3, T2 of the water ^1^H signal was determined from axial images from the belly of the calf musculature. T2 was calculated from a series of images generated at 1.5 T by a multi-spin-echo sequence with 10 equidistance echo times from 13 to 130 ms. Further parameters are 90° flip angle, a suppression of the fat signal, a repetition time of 1600 ms, a pixel matrix of 256 × 256 at 1 mm × 1 mm pixel size and a slice thickness of 5 mm. An image showing the sum of intensities of the same series of T2 images was used to manually outline the areas of the SOL, GL and GM. These areas were transferred to the T2 parameter image to calculate the mean T2 value of each muscle. The Gaussian distribution of T2 values among all pixels was then visualized in a spectrum-like diagram, and the mean time constant T2 (or transversal spin-relaxation) from all pixels in the selected region of interest were then calculated with ImageJ software.

### Fatigue test

The fatigue resistance of the plantar flexors was tested on BDC-6, *R* + 1 and at *R* + 28. The measurements were performed in the semi-recumbent position as described previously (Zange et al. 2008). In short, the participants laid on their back with the upper body elevated by 30° and supported by triangular padding. Their legs were extended in a 40 cm-diameter bore of a nuclear magnetic resonance (NMR) magnet (Bruker-Biospec 47/40, Bruker-Medical, Ettlingen, Germany). The upper body, arms and head were outside the magnet. The right calf was placed on a calf holder with an integrated NMR surface coil and a pair of NIRS optodes in the centre of the magnet. The knee of the right leg was bent about 10° and the foot was fixed with belts on a pedal. The pedal was connected to a load via pulley system, where the ankle could move between 70 and 40° relative to the horizontal. At baseline, the participants executed a dynamic exercise task against a load corresponding to 50% body mass on the day of testing.

To establish intrinsic changes in the fatigue resistance of the muscle, unrelated to loss of muscle mass during bed rest, we adjusted the load to be lifted by the plantar flexors in proportion to the change in plantar flexor muscle size during bed rest, to ensure that the load per muscle cross-sectional area was similar before and after bed rest. Therefore, in the subsequent sessions we decreased the load in proportion to the decline in muscle cross-sectional area that was estimated by pQCT. Therefore, following bedrest and at *R* + 28, for each subject the weight to be lifted (LW) was calculated as the product between the weight lifted at baseline and the ratio between CSA_PF_ in bed rest (or *R* + 28) and CSA_PF_ at baseline as follows:

$${\text{LW}}_{{({\text{BR}})}} = {\text{LW}}_{{({\text{BL}})}} \times \left( {{\text{CSA}}_{{{\text{PF}}\left( {{\text{BR}}} \right)/}} {\text{CSA}}_{{{\text{PF}}({\text{BL}})}} } \right).$$

Descriptive data of the lift weights applied for the test before (BL) and after bedrest (*R* + 1 and *R* + 28) for each subject are reported in Table 1.

**Table 1:** Descriptive data of the lifted weights before (BL) and after bed rest (*R* + 1) and recovery from bed rest (*R* + 28) for each subject who participated in the study

Subject	BL	*R* + 1	Relative change between BL and *R* + 1	*R* + 28	Relative change between BL and *R* + 28
	Lift weight (kg)	Lift weight (kg)	Δ change% Lift weight (kg)	Lift weight (kg)	Δ change% Lift weight (kg)
A	40.60	33.81	− 16.71	39.96	− 1.58
B	37.14	31.77	− 14.37	36.88	− 0.58
C	40.60	34.38	− 15.32	39.54	− 2.62
D	33.10	27.86	− 15.83	33.35	0.75
E	36.22	29.21	− 18.87	37.23	3.42
F	43.00	36.59	− 14.90	41.42	− 3.66
G	38.35	31.78	− 17.24	38.46	0.16
H	38.12	33.21	− 12.84	38.63	1.40
K	38.40	33.96	− 11.55	38.91	1.34
L	38.20	31.39	− 17.82	39.73	4.00

The exercise task started with 1 min rest. Subsequently, the participants executed 8 bouts of 24 maximal concentric plantar flexions (each bout lasting 30 s), interposed by seven 20-s rest periods. A final 300-s rest period completed the protocol. Participants were guided in their contractions by a metronome and a digital clock and received verbal encouragement. Following each concentric contraction, the weight was dropped passively. Data were obtained and analysed using custom-built software on the software platform DASYLAB 7.0 (National Instruments, Mönchengladbach, Germany). For each contraction, contraction velocity, power and surface electromyography (sEMG) were recorded. All parameters were averaged for each exercise bout (further on called exercise interval). Contraction velocity, power (given as the product of force and velocity) and sEMG activity (root mean square, RMS signals) were then expressed as % of the first exercise interval. The int8/int1 ratio was indicative of the degree of fatigue developed.

### Surface electromyography

A bipolar surface electromyogram (sEMG) was recorded from the soleus muscle. Since the NMR coil was placed under the belly of the calf, the EMG electrodes were placed at the distal part of the lower leg to avoid technical disturbance between the MRS and the EMG measurements. At the distal part of the lower leg is located only the soleus muscle. The electrodes were placed at 2/3 of the distance between the medial condylus of the femur and the medial malleolus. The inter-electrode distance was 2 cm, and the reference electrode was placed at the shin over the tibia. The EMG signal was conducted by shielded cables that ran from the magnet to a preamplifier, resistant to the NMR pulses (RWTH-Aachen, Germany), that pre-amplified the signal 1000-fold. The signal was recorded at a 1000-Hz sampling rate and digitally band-pass filtered between 10 and 400 Hz. Each concentric phase was analysed for amplitude of the root mean square (RMS).

### Near infrared spectroscopy

Changes in the concentrations of oxygenated and deoxygenated haemoglobin and myoglobin in calf muscles were obtained with a continuous-wave near-infrared spectrophotometer (NIRS; Oxymon, Artinis Medical Systems, Andelst, NL) that generates light at wavelengths of 905, 850 and 770 nm. The NIRS optodes were integrated in a calf holder that also housed the radio-frequency surface coil for the ^31^P NMR (see below). The optode distance was 40 mm. The NIRS measured all the three parts of the calf muscle. Data were sampled at a frequency of 10 Hz. Due to overlap of the spectra, it is impossible to distinguish between haemoglobin and myoglobin. For simplification we regard the changes in the NIRS signal to reflect changes in the oxygenated and the deoxygenated state of haemoglobin only. The modified Lambert–Beer law that incorporates a differential path-length factor of 4.6 to correct for scattering of photons in the tissue was used to convert the changes in absorption at the discrete wavelengths into µM concentration changes of tetramers of HbO_2_ and HHb in the tissue. The sum of HbO_2_ and HHb represented the changes in total tissue content of haemoglobin (tHb). All signals were baseline corrected relative to the first 5 s of data prior to initiation of the exercise. Maximum or minimum values during the exercise period were used to determine absolute concentration changes for desoxyhaemoglobin ([Δ]HHb), oxyhaemoglobin ([Δ]HbO_2_)  and total haemoglobin ([Δ]tHb).

### ^31^P magnetic resonance spectroscopy and energy metabolism

The NMR spectra of the calf were obtained in a 4.7-T 40-cm horizontal-bore spectrometer (Bruker-Biospec 47/40, Bruker-Medical, Ettlingen, Germany) using a 5 cm-diameter ^1^H/^31^P surface coil placed under the belly of the right calf. The NMR measured all the three parts of the calf muscle. The resonance frequencies were 200 MHz for ^1^H and 81 MHz for ^31^P. ^1^H spectra were used to optimize magnetic field homogeneity (shimming). Pulses of 100-μs duration and 60° flip-angle at the centre of the coil were used for ^31^P NMR spectroscopy and were given during the resting phase following each contraction in a single bout. Three spectra were obtained for each 30-s exercise interval and two spectra were obtained for each 20-s resting interval. Spectra were evaluated for PCr, Pi, ATP and PME. The intracellular pH (pHi) was determined by the chemical shift of the phosphate peak in ATP (δ in ppm) relative to PCr (Taylor et al. 1983):

$${\text{pHi}} = 6.75 + \log \left( {\left( {\delta - 3.27} \right)/\left( {5.69 - \delta } \right)} \right).$$

The mean PCr signal of six baseline spectra was set to 100%. All following PCr values and the intensities of all other metabolites were expressed as % initial PCr.

### Cardiopulmonary parameters during a maximal oxygen uptake test

Oxygen consumption, carbon dioxide emission and respiratory exchange ratio were recorded breath-by-breath with a Metalyzer spirometer (CORTEX Metalyzer; CORTEX Biophysik, Leipzig, Germany). The electrocardiogram, ventilation, respiratory exchange ratio and heart rate were monitored throughout the test. The blood pressure was monitored with a Finometer (Biopac Systems, Goleta, CA). Maximal oxygen uptake (V'O_2max_) was assessed using a graded exercise protocol on an electronically braked cycle ergometer (Excalibur Sport; Lode, Groningen, The Netherlands). Participants were considered to have reached V'O_2max_ if they fulfilled at least two of the following three criteria: they had reached the predicted maximal heart rate; they had a respiratory exchange ratio > 1; they could not maintain the cadence of 60 revolutions·min^−1^ because of exhaustion (Bosutti et al. 2016).

### Statistics

A repeated-measures ANOVA was performed for the changes in the parameters during the fatigue test, with as within factors time (BL, *R* + 1, *R* + 3, *R* + 14, *R* + 28), state (contraction or recovery phase during the test) and interval (8 intervals), and group (BR vs. NUTR) as between factor. If a main effect of interval and/or an interaction was found, Bonferroni-corrected post-hoc tests were performed to locate the differences. Bed rest-induced changes in all other parameters were assessed with a two-way ANOVA with as factors time (BL vs. HTD + 21) and group (BR *vs.* NUTR). Three-way interactions were excluded. Regression analysis (GraphPad Prism 8 Software, U.S.A.) of individual data was performed to analyse relationships between selected variables. Statistical analysis was performed with SPSS (IBM SPSS, USA). Statistical significance was set at *P* < 0.05**.** Values are reported as mean ± standard deviation (SD).

## Results

### Plantar flexor muscle size and water content

**Fig. 1:**
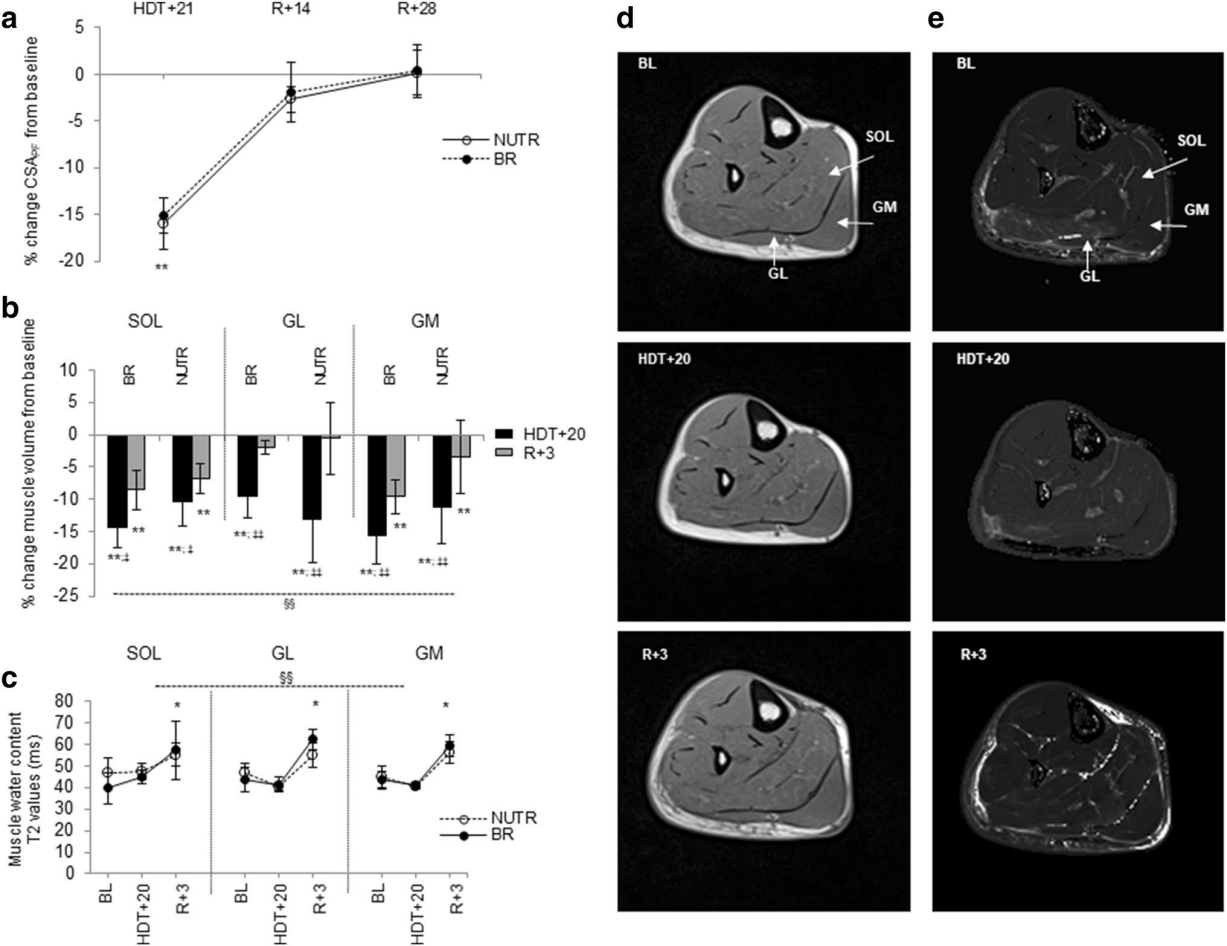
Effects of 21 days of bed rest (BR) and WP + KHCO_3_ supplementation (NUTR) on plantar flexor muscle mass. **a** Plantar flexor muscle cross-sectional area (CSA_PF_) assessed by pQCT. Data are presented as % change CSA_PF_ from baseline **: HDT + 21 < BL, *R* + 14 and *R* + 28 at *P* < 0.001. **b** Plantar flexor muscle volume assessed by MRI. Data are presented as % change muscle volume from baseline. Differences between muscle type: ^§§^SOL > GL > GM at *P* < 0.001. Condition effect: SOL: **HDT + 20 and *R* + 3 < BL at *P* < 0.001; ^‡^HDT + 20 < *R* + 3 at *P* = 0.023. GL: **HDT + 20 < BL at *P* < 0.001; ^‡‡^HDT + 20 < *R* + 3 at *P* = 0.005. GM: **HDT + 20 and *R* + 3 < BL at *P* < 0.01; ^‡‡^HDT + 20 < *R* + 3 at *P* = 0.007. **c** Changes in muscle water content assessed by MRI. The images showed T2 (or transversal spin-relaxation)—values given in milliseconds (intensity = T2). Differences between muscle type: ^§§^SOL > GL > GM at *P* < 0.001. Differences between condition at p < 0.001: *R* + 3 > HDT + 20, BL at *P* < 0.001, irrespective of muscle type. There were no significant NUTR effects. Data are expressed as means ± SD. **d** Representative MRI images and **e** T2 images (merge of six images) of the left lower leg of the same subject before bed rest (BL), after 20 days of bed rest (HDT + 20) and at 3 days after re-ambulation (*R* + 3). SOL, GM and GL muscles are shown by arrows

Bed rest, irrespective of WP + KHCO_3_ supplementation, induced a loss of muscle volume as reflected by the decrease in CSA_PF_ (Fig. 1a; *P* < 0.001) and decrease in volume of the SOL, GL (*P* < 0.001) and GM (*P* < 0.01) (Fig. 1b). The loss of muscle volume was partly recuperated just after 3 days (Fig. 1b and panel d) and did not differ significantly from BL at 14 days after completion of bed rest (Fig. 1a). The rapid volume recovery within the first days after bed rest was predominantly caused by an intracellular uptake of water by the muscle, as suggested by the increase in T2 at *R* + 3 in all muscles (Fig. 1c and panel e). No significant differences in T2 were found between pre and post bed rest, with or without whey supplement, in terms of T2-relaxation time (Fig. 1c). Therefore, the immobilization did not alter the composition of PF muscles in terms of free *vs.* bond water.

### Plantar flexor muscle fatigue

**Fig. 2:**
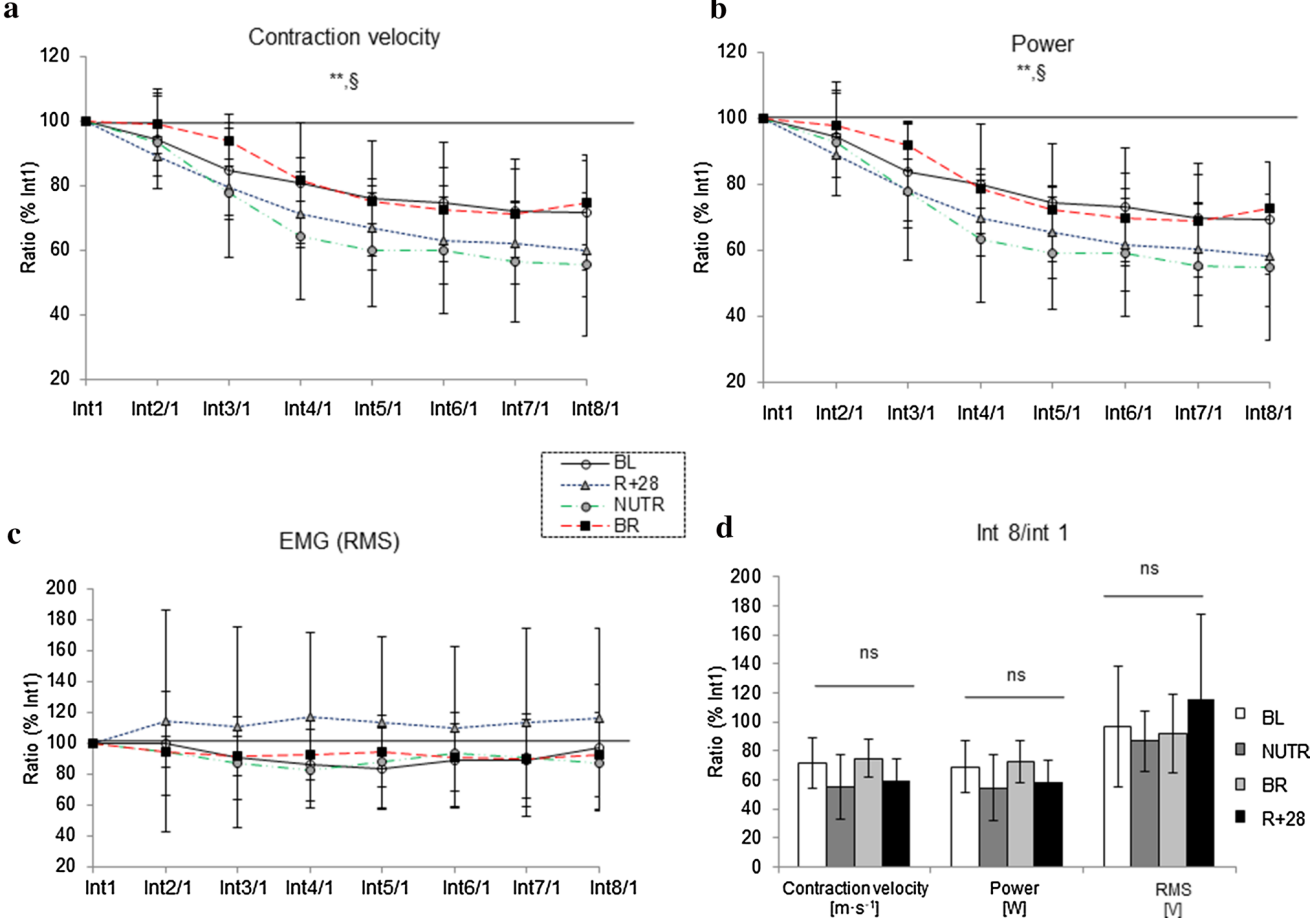
Contraction velocity, mechanical power and electromyographic (EMG) activity during the fatigue test: **a** contraction velocity, **b** power and **c** soleus EMG activity (root mean square (RMS)) expressed as % of the first exercise interval and **d** the interval 8: interval 1 ratio as a measure of fatigue. **Significant difference between final exercise interval and initial exercise interval, at *P* < 0.001, irrespective of condition. §BR > NUTR at *P* < 0.05. BL: baseline; NUTR: bed rest plus WP + KHCO_3_; BR: bed-rest with standardized diet; *R* + 28: recovery period. Data are expressed as mean ± SD

To assess the intrinsic muscle fatigue resistance, independent of muscle mass, we executed a dynamic exercise task before and after bed rest where the initial load: CSA_PF_ ratio was similar to BR at *R* + 1 and *R* + 28 (lifted weights applied for BL, *R* + 1 and *R* + 28 are reported in Table 1). Muscle velocity and power (Fig. 2a, b) were lower in the last (interval 8) than in the initial (interval 1) interval of the exercise test, irrespective of condition (*P* < 0.001), but there were no significant changes in sEMG activity (RSM % with respect to the 1st interval) of the SOL (Fig. 2c) during the test. The int8/int1 ratio did not differ significantly for velocity, power and RMS between BL, *R* + 1 and *R* + 28, or between BR and NUTR groups (Fig. 2d).

Representative force, lift and EMG recordings registered from a subject during the exercise test at BL are shown in Supplementary Fig. 1.

### Muscle tissue oxygenation

**Fig. 3:**
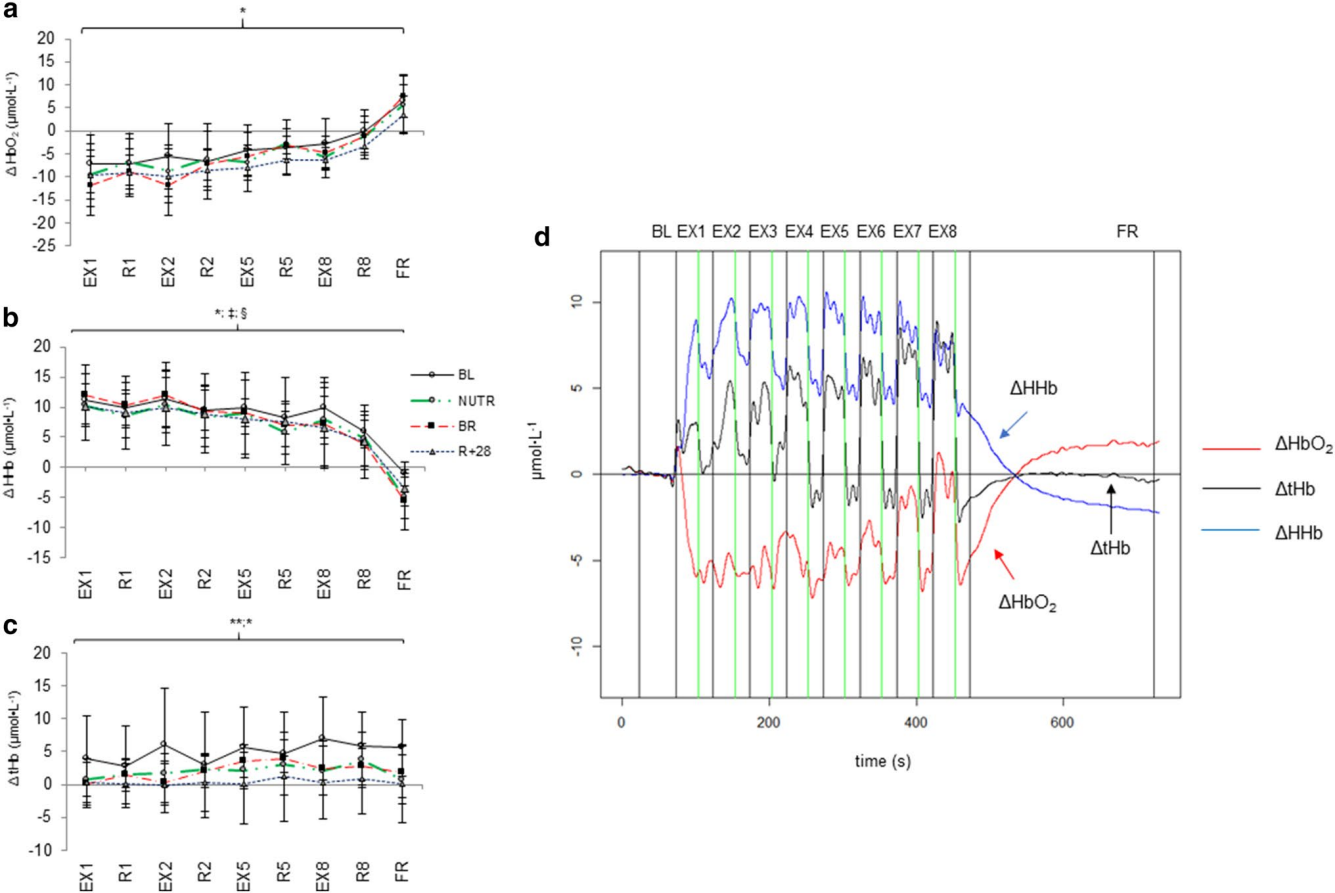
ΔOxy-haemoglobin (ΔHbO_2_) (**a**), ΔDesoxy-haemoglobin (ΔHHb) (**b**) and ΔTotal-haemoglobin (ΔtHb) (**c**), across intervals of the fatigue test. **a** ΔHbO_2_: *BL > BR, *R* + 28 at *P* < 0.01 in exercise (EX) phase. **b** ΔHHb; *BL > *R* + 28 at p = 0.039 in exercise (EX) phase; §BL > BR at *P* = 0.018 in relaxation (*R*) phases of the test; ‡ BL > BR at *P* = 0.025 at final recovery (FR). **c** ΔtHb: **BL > BR, *R* + 28 at *P* < 0.001 and *R + 28 < BR, at *P* = 0.014 in exercise (EX) phase of the test. BL: baseline; **d** Representative NIRS recording from a subject during the exercise task; NUTR: bed rest plus WP + KHCO_3_; BR: bed-rest with standardized diet; *R* + 28: recovery period (28 days after completion bed rest). Data are expressed as mean ± SD

Irrespective of condition, the ΔHbO_2_ concentration in the calf muscles was reduced at the start of the test and remained reduced during the progression of the test (Fig. 3a), where ΔHHb showed a trend (not significant) for the opposite pattern (Fig. 3b) even though ΔtHb was slightly elevated during the test (Fig. 3c).

During the contraction phases, muscle ΔHbO_2_ levels were lower (*P* = 0.002) in overall bed rest condition (HDT + 21) and at *R* + 28 compared to pre-bed rest (data not shown). No significant differences were found between BR and NUTR groups (Fig. 3a). In addition, the ΔtHb during the contraction phases at any moment in the fatigue test was lower (*P* < 0.001) after bed rest and at *R* + 28, indicating an attenuated contraction-induced increase in blood flow (Fig. 3c). Bed rest also affected the recovery of ΔHHb (Fig. 3b) either between contraction interval (*P* = 0.018) or at final recovery after completion of exercise test (*P* = 0.025). Finally, the restoration of blood flow after the contraction phases did not significantly differ between BR and NUTR groups (Fig. 3c). Raw example for NIRS recordings during the exercise task is shown in Fig. 3, panel d.

### Energy metabolism

**Fig. 4:**
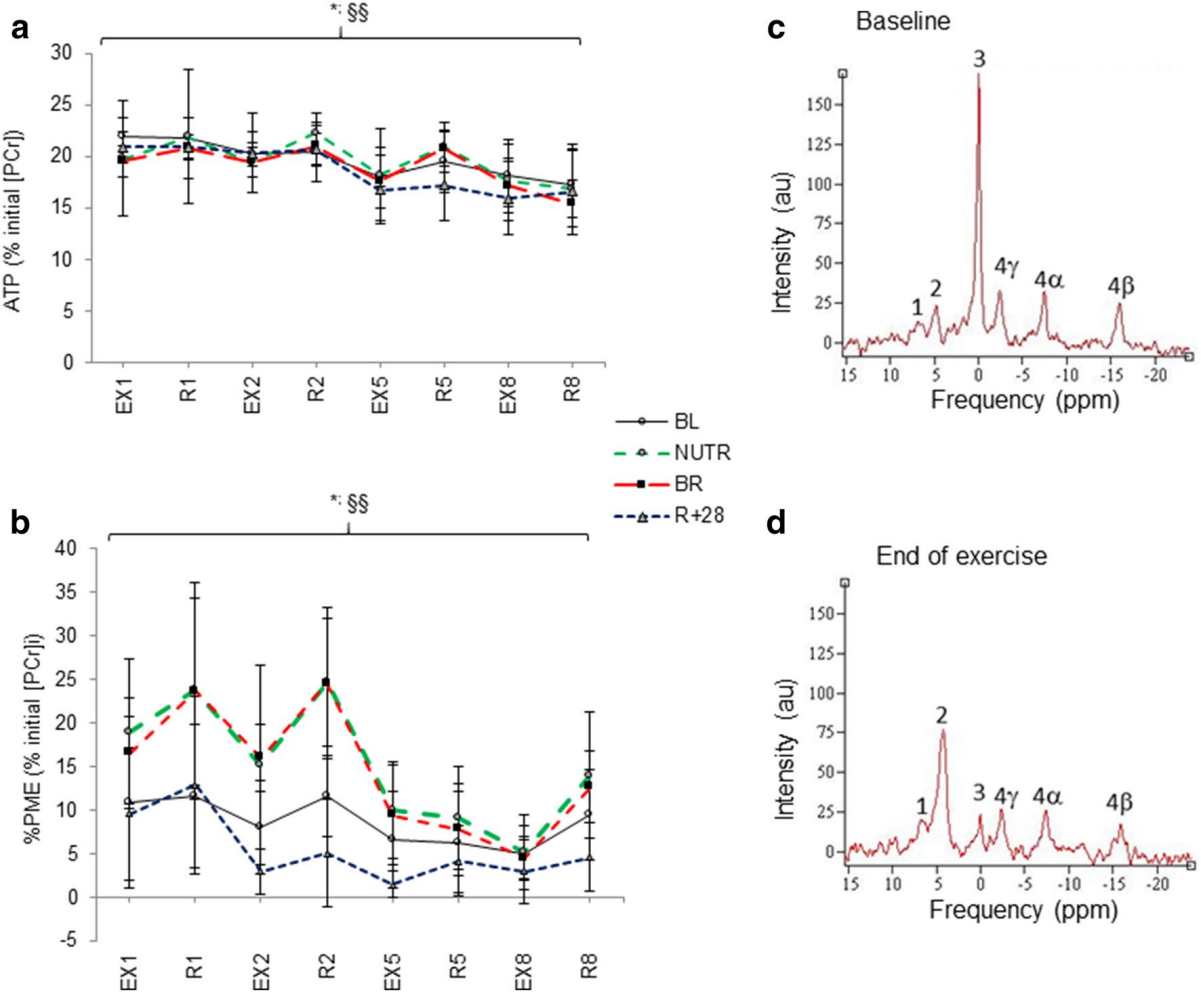
Time course changes in ATP (**a**) and PME (**b**) in the calf musculature during the fatigue test: Panel **a**: ^§§^differences between intervals, at *P* < 0.001; *intervention effect (NUTR) at *P* = 0.034 and intervention *exp time interaction, indicating a higher ATP in NUTR than BR at rest. Panel **b**: *R + 28 differences between EX and *R* phases (EX < *R*) at *P* < 0.01; ^§§^post bed rest: differences between intervals in *R* phase (2 > 8), at *P* < 0.001. Panels show data at interval 1, 2, 5 and 8. Panels **c** and **d** show representative NMR spectra recorded from a subject before and after exercise (1 = PME; 2 = P_i_; 3 = PCr; 4αβγ = ATP phosphate groups); au = arbitrary units; ppm, Parts/million. Data are expressed as mean ± SD

Representative NMR spectra recorded before and after exercise from a subject are shown in Fig. 4c, d.

Irrespective of condition, the development of muscle fatigue during the exercise test was associated with reduced intramuscular ATP (Fig. 4a) and PCr levels (*P* < 0.001; Supplementary Fig 2a). This was accompanied by a rise in Pi and a reduction in pHi (*P* < 0.001; Supplementary Fig. 2b, c). There was, however, no significant effect on the rate of PCr formation, as indicated by unchanged τ (time constant of PCr increase in s: BL: 108.0 ± 5; BR: 107.5 ± 10; *R* + 28: 76.6 ± 36; ns).

**Fig. 5:**
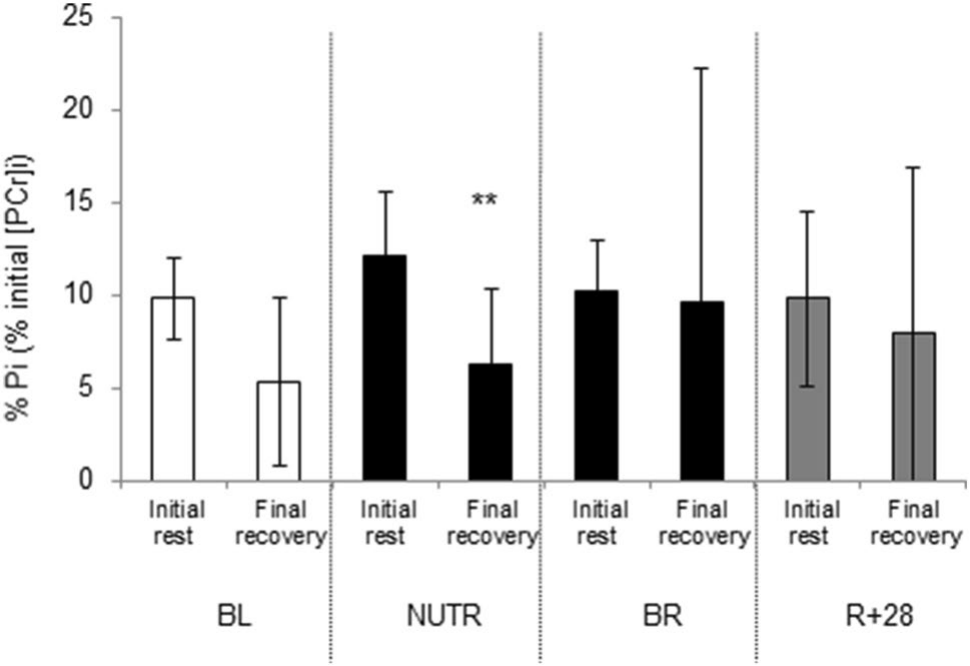
Time effect of WP + KHCO_3_ supplementation on P_i_ intramuscular levels. *Final recovery < initial rest at *P* < 0.001 with countermeasure * condition interaction at *P* < 0.05, reflected by attenuated rise of Pi in NUTR group compared to BR during the fatigue test. Data are expressed as mean ± SD

Whey supplementation increased intramuscular ATP levels in all phases of the exercise test (*P* = 0.034; Fig. 4a) and reduced (*P* < 0.05; Fig. 5) the overall Pi accumulation at the end of the final recovery phase. Bed rest, irrespective of whey supplement, induced a larger increase (*P* < 0.001) in PME concentration than at baseline and *R* + 28 during the recovery phases of the exercise test (Supplementary Fig. 3).

### Cardiopulmonary parameters

Peak values of the main cardiopulmonary and gas-exchange variables are shown in Table 2. The respiratory exchange ratio and peak heart rate were similar before and after bed rest, indicating that the subjects went to their maximum in both instances. Whole body aerobic capacity and peak power were reduced following bed rest (*P* < 0.01), irrespective of receiving WP + KHCO_3_ supplement or not. This was accompanied by a reduction (*P* = 0.001) in V'O_2_ per heartbeat, indicative of a reduced stroke volume and/or impaired oxygen extraction.

**Table 2:** Peak values of the main cardiopulmonary variables determined during an incremental bicycle exercise test before (BL) and after bed rest (*R* + 1)

	BL (BDC-7)	*R* + 1 (standardised diet)	*R* + 1 (NUTR)
Peak HR (beats·min^−1^)	187 ± 7	192 ± 3	193 ± 7
RER (V'CO_2_/V'O_2_)	1.27 ± 0.07	1.28 ± 0.10	1.30 ± 0.14
VE peak (L·min^−1^)	160 ± 20	163 ± 29	171 ± 19
V'CO_2max_ (mL·min^−1^)	4.39 ± 0.46	3.97 ± 0.62*	4.20 ± 0.36*
V'O_2max_ (mL·min^−1^)	4.03 ± 0.58	3.32 ± 0.64*	3.51 ± 0.67*
Power (W)	278 ± 51	236 ± 62*	257 ± 47*
V'O_2max_/BMI (mL·kg^−1^·min^−1^)	53 ± 6	45 ± 6*	46 ± 8*
V'O_2_/beat (mL·beat^−1^)	22 ± 3	17 ± 3**	18 ± 3**

Data are mean±SD. BL = before bed rest (BDC−7), *R*+1 = bed rest. ** *P*=0.001; *P<0.01 vs BL

*BMI* body max index, *HR* heart rate, *RER* respiratory exchange ratio, *VE* ventilation, *V′O*_*2*_ oxygen consumption, *V′O*_*2*_*max* maximal oxygen uptake, *V′CO*_*2*_ carbon dioxide emission

## Discussion

The major question addressed in the present study was whether 21 days of bed rest did induce a reduction in plantar flexor muscle fatigue resistance during a series of shortening contractions and to what extent any decrement could be explainable by altered energy metabolism and muscle oxygenation. In addition, the effects of WP + KHCO_3_ supplementation during bed rest on muscle fatigue resistance were investigated.

The principal observation of this study is that, in contrast to our hypothesis, the intrinsic fatigue resistance of skeletal muscle during a series of repetitive shortening contractions was not affected by 21 days of bed rest or diet-intervention. Bed rest induced a shift in muscle metabolism towards glycolysis, as suggested by the marked increase in muscle PME concentrations during the exercise test following bed rest, but without affecting other energy metabolism parameters significantly. In addition, there was some indication of an impaired oxygen extraction, which corresponds to the reduced succinate dehydrogenase activity seen in the same subjects in our previous study (Bosutti et al. 2016).

### Changes in muscle volume and T2

A large part of the changes in mechanical muscle function seen in bed rest or space flight are attributable to the loss of muscle volume. However, numerous space flight or bed rest studies show a larger reduction in muscle strength compared to the loss of muscle volume (Lee et al. 2000; Trappe et al. 2009; Akima et al. 2000; Alkner and Tesch 2004; LeBlanc et al. 1988). There are also alterations in muscle water content due to the fluid shift that accompanies (head-down-tilt) bed rest and space flight (Greenleaf et al. 1977; Rittweger et al. 2013; Smith et al. 1997). In bed rest, muscle dehydration consequent to the fluid shift could significantly affect muscle force development, since hydration of actomyosin is required for cross-bridge cycling and force generation (Oplatka 1989; Yamada et al. 2017). Dehydration has also been found to be associated with muscle damage in power athletes (Isik et al. 2018), and if it occurs also in bed rest it may contribute to lower muscle strength.

However, we did not find any indication of a change in hydration status after 20 days of bed rest (Fig. 1c). As shown by the lack of changes in T2—relaxation time, muscle hydration was elevated at 3 days after completion of bed rest. This increased hydration was probably attributable to the re-distribution of fluids occurring after cessation of bed rest and may be the main cause of the increase in muscle size observed after just 3 days of returning to the up-right condition. Accordingly, it is reported that the fluid shift in microgravity is quickly restored and accumulates in the lower extremities, even just a few hours after re-ambulation (Leach et al. 1996; LeBlanc et al. 1987, 1992, 2000).

It is assumed that the interstitial space may shrink more markedly than the intracellular fluid space during bed rest (Rittweger et al. 2013) and if so, it may explain why bed rest was not accompanied by fiber atrophy (Bosutti et al. 2016; Blottner et al. 2014) despite a significant loss in muscle mass. Likely, adaptive compensatory mechanisms such as an increased expression of aquoporin-4 (Basco et al. 2010, 2013; Frigeri et al. 2004) helped maintain fibre size and integrity during the bed rest-induced fluid shift.

### Muscle fatigue resistance

Bed rest did not cause a reduction in muscle fatigue resistance during repeated shortening contractions. Indeed, this finding was not in agreement with the impaired muscle performance commonly observed post-flight, or after prolonged bed rest (Petersen et al. 2017; Kawakami et al. 2001; Mulder et al. 2007). It is interesting to note, however, that the repeated contractions in the fatigue test were accompanied by the absence of any difference in EMG amplitude before and after bed rest. This endorses the idea that neuromuscular activation during the fatigue test was not affected by 21-day bed rest and could explain at least in part the absence of a decreased fatigue resistance found in our study. Something similar was also described in another model of muscle disuse, where both fatigability and EMG median frequency kinematics in the calf musculature were not affected by muscle unloading induced by orthosis (Weber et al. 2014).

### Muscle fatigue and metabolism

The fatigue development during contractions could be due to a diminished formation of actin-myosin cross-bridges (Bigland-Ritchie et al. 1986; Tesch et al. 1990). It is plausible that the progressive accumulation of P_i_ and H^+^ (Robertson and Kerrick 1979) during our fatigue test (Supplementary Fig. 1, 2) slowed the rate of cross-bridge cycling (Jones et al. 2009). Nonetheless, the accumulation of Pi and decrease in pH during the fatigue test was similar before and after bed rest, indicating that energy metabolism was not affected by 21 days of disuse. It is important to note that whey supplementation reduced the overall Pi accumulation at the end of the final recovery phase, which may suggest that the whey supplement resulted in a faster muscle recovery after exercise (Fig. 5). Compared to the standard diet, the whey diet increased intramuscular ATP levels in all phases of the exercise test (Fig. 4). Although still speculative, this might suggest a higher contribution of oxidative metabolism to ATP production, when bed rest is supported by whey-intervention, in line with our previous observation in the same participants that whey protein attenuated the bed rest-induced reduction in succinate dehydrogenase activity (Bosutti et al. 2016).

The only marked difference we observed in bed rest was a more pronounced increase of PME concentrations, particularly at the onset of the fatigue test. The main PMEs are glucose-6-phosphate and fructose-6-phosphate, two intermediates in glycolysis (Hunter et al. 2006). Their accumulation during exercise could indicate a mismatch in the activity of phosphorylase and phosphofructokinase (Ren and Hultman 1985). Another possible cause may be metabolic inflexibility, an impaired ability to switch from fatty-acid to glucose oxidation, during transition from a fasted to a fed state, seen in the same subjects as in our study during transition from a fasted to a fed state after bed rest (Rudwill et al. 2018). This metabolic inflexibility may hamper the transition to a greater reliance on carbohydrate metabolism at the onset of exercise and explain the greater rise in muscle PME during contractile activity after bed rest (Muoio 2014). Yet, accumulation of PME did not result in a faster accumulation of Pi, or depletion of PCr and ATP. Our results together with others (Rudwill et al. 2018; Hunter et al. 2006) further corroborates that 21 days of bed rest induced a shift to a larger reliance on glycolysis.

### Impact of aerobic capacity, cardiopulmonary function and muscle oxygenation on muscle performance

The reduction in V'O_2max_ is most likely attributable to both a decline in stroke volume, as reflected by the smaller oxygen pulse, and a reduction in oxidative capacity of the muscle (Bosutti et al. 2016). Initially, reductions in stroke volume may be a consequence of decrements in plasma volume during immobilization, with the majority of the losses occurring in the first few days of unloading (Greenleaf et al. 1977; Fortney et al. 1991; Zorbas et al. 1999). Whatever the cause, the cardiovascular and muscle adaptations during bed rest or spaceflight may not follow the same time course. For example, it is reported that after 35 days of bed rest, the impaired skeletal muscle oxidative metabolism during dynamic knee extension is largely due to an impairment of muscle oxidative function, substantially excluding any cardiovascular limitations to O_2_ delivery (Salvadego et al. 2011).

In addition, an impaired vasodilatation after disuse (Thijssen et al. 2010) may diminish the convective delivery of oxygen to the working muscle and result in decreased fatigue resistance (Ade et al. 2015). In fact, at any moment during the fatigue test, muscle ΔHbO_2_ levels were lowered after bed rest and this was in agreement with the lower oxidative capacity we previously reported in the same study (Bosutti et al. 2016). Accordingly, we also found that bed rest was accompanied by a marked reduction in V'O_2max_ per heartbeat, which is indicative of a possible impaired oxygen extraction by muscle tissue. Indeed, bed rest reduced the concentrations of the ΔtHb and ΔHbO_2_, which could imply that PF muscles began more ischemic and consequently more prone to turn to anaerobic energy sources, following 21-day bed rest.

## Limitations

The load in the fatigue test was normalized to the CSA, which does not consider potential neural adaptations during bed rest. Such adaptations cannot be ruled out by sEMG. In addition, the sEMG was derived from the soleus only, while force is measured from the triceps surae.

## Conclusions

In conclusion, despite the significant reduction in plantar flexor muscle volume, 21 days of bed rest did not impair the intrinsic fatigue resistance of plantar flexor muscles during concentric exercise. There were some signs of a shift in muscle metabolism toward glycolysis and an impaired muscle oxygen extraction. Notably, the muscle volume loss caused by bed rest was partly recuperated after just 3 days of re-ambulation. This rapid gain was predominantly caused by fluid redistribution to muscle. As the bed rest-induced decline in muscle oxidative capacity is attenuated when combined with whey protein supplementation (Bosutti et al. 2016), we expected a bed rest-induced decrease in muscle fatigue resistance attenuated by whey protein supplementation. This was not found and confirms that in whole muscle, changes in fatigue resistance may be dissociated from changes in the oxidative capacity (Degens and Veerkamp 1994).

## Electronic supplementary material

Below is the link to the electronic supplementary material.

Supplementary Fig. 1 Representative force, lift, and EMG recordings registered from a subject during the exercise test. a,c,e Images show, respectively, force, lift and EMG signals recorded during the exercise test. **b,d,f** Images show a zoomed 30-sec part of the same exercise interval (TIF 185 kb)

Supplementary Fig. 2 Time course changes in PCr **a**, Pi **b** and pHi **c** in the calf musculature during the fatigue test. §§ differences between intervals, at *P*<0.001. No condition effects. Data are expressed as mean ± SD (TIF 110 kb)

Supplementary Fig. 3 The overall effect of bed rest on PME levels in the calf musculature during the fatigue test. **Condition effect at *P*<0.001. §Post bed rest data only recovery: Interval *P*<0.001; 2>5-8 at *P*≤0.015. BL: baseline; HDT+21: 21-day' bed rest (mean with/without WP+KHCO3); R+28: mean recovery period. The panel shows data at interval 1,2,5 and 8. Data are expressed as mean ± SD (TIF 60 kb)

## Abbreviations

ANOVA: Analysis of variance
ATP: Adenosine triphosphate
BDC: Days before bed rest phase
BL: Baseline (pre bed rest phase)
BR: Bed rest (post bed rest phase)
CSA_PF_: Plantar flexor muscle cross-sectional area
DLR: Deutsches Zentrum für Luft-und Raumfahrt
EMG: Electromyography
ESA: European Space Agency
EX: Contraction phases (exercise test)
FR: Final recovery (exercise test)
GL: *M.**gastrocnemius lateralis*
GM: *M.** gastrocnemius medialis*
Hb: Haemoglobin
HbO_2_: Oxyhaemoglobin
HHbO_2_: Deoxyhaemoglobin
HDT: Head-down tilt
MRI: Magnetic resonance imaging
NIRS: Near infrared spectroscopy
NMR: Nuclear magnetic resonance
NUTR: Nutritional intervention
PCr: Phosphocreatine
PF: Plantar flexor muscles
Pi: Inorganic phosphate
PME: Phosphomonoesters
pQCT: Peripheral quantitative computed tomography
*R*: Recovery (recovery phases during the exercise test)
*R*+ _(__*n*__)_: Recovery phase (_(__*n*__)_ days after completion of bed rest)
RER: Respiratory exchange ratio
ROI: Region of interest
SD: Standard deviation
SOL: *M.**soleus*
T2: Transverse spin relaxation
tHb: Total haemoglobin
VE: Ventilation
V′O_2_: Oxygen consumption
V′O_2__max_: Maximal oxygen uptake
V′CO_2_: Carbon dioxide emission
WP + KHCO_3_: Whey protein (WP) plus potassium bicarbonate (KHCO_3_)
