# Supplementary figures and images for: Effects of 21 days of bed rest and whey protein supplementation on plantar flexor muscle fatigue resistance during repeated shortening contractions
Source: Eur J Appl Physiol. 2020 Mar 4;120(5):969–83. doi: 10.1007/s00421-020-04333-5 (PMC7181505; doi:10.1007/s00421-020-04333-5)

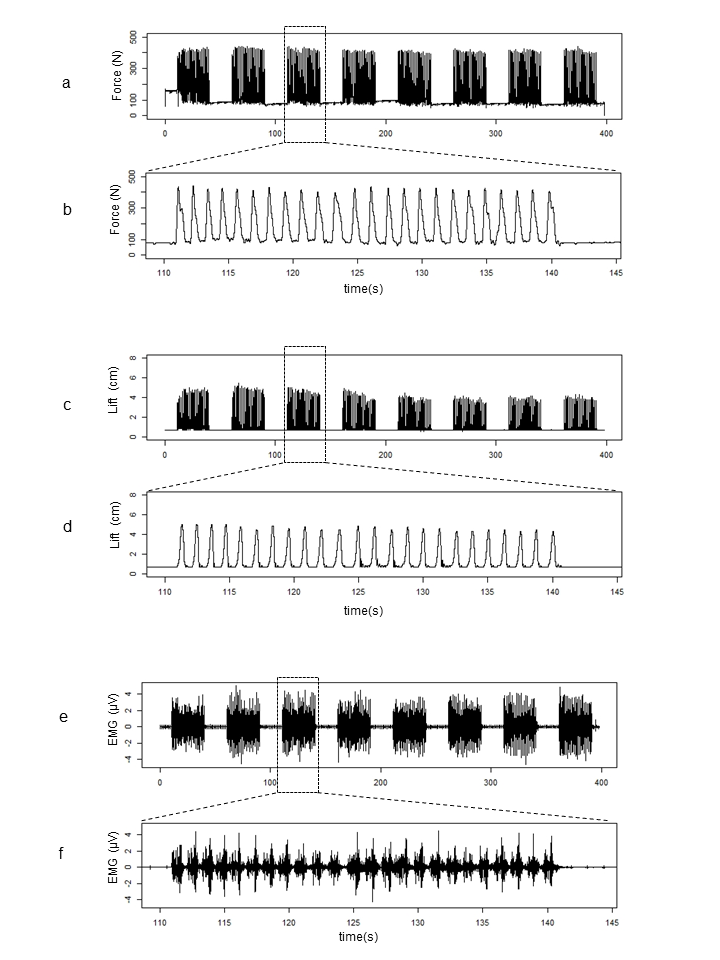

Supplement: Supplementary file 1 — Supplementary Fig. 1 Representative force, lift, and EMG recordings registered from a subject during the exercise test. a,c,e Images show, respectively, force, lift and EMG signals recorded during the exercise test. b,d,f Images show a zoomed 30-sec part of the same exercise interval (TIF 185 kb) [file 421_2020_4333_MOESM1_ESM.tif]

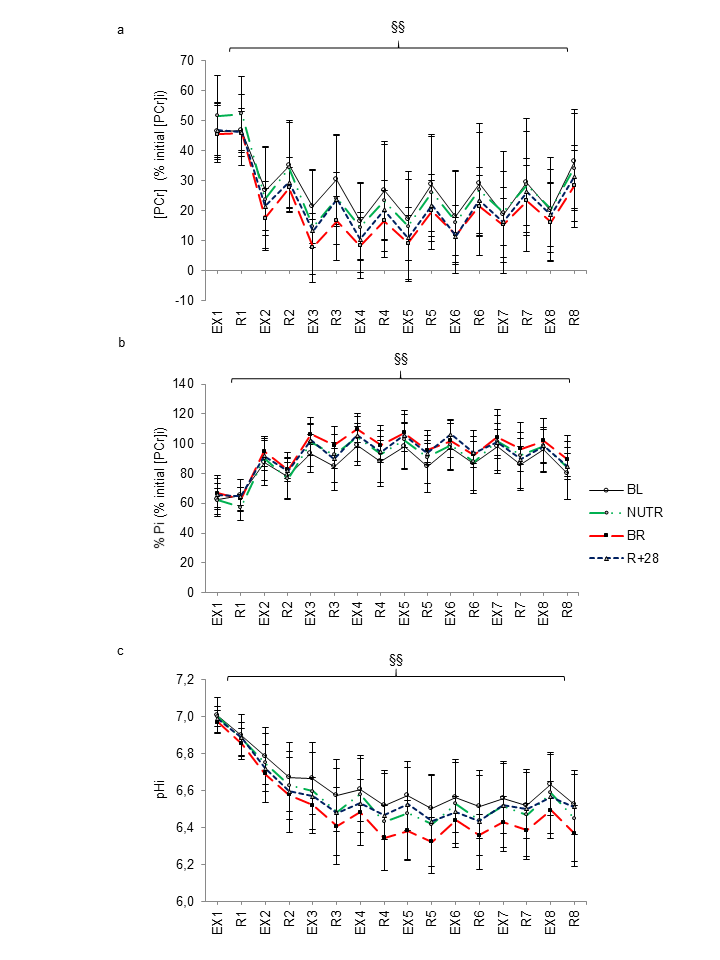

Supplement: Supplementary file 2 — Supplementary Fig. 2 Time course changes in PCr a, Pi b and pHi c in the calf musculature during the fatigue test. §§ differences between intervals, at P<0.001. No condition effects. Data are expressed as mean ± SD (TIF 110 kb) [file 421_2020_4333_MOESM2_ESM.tif]

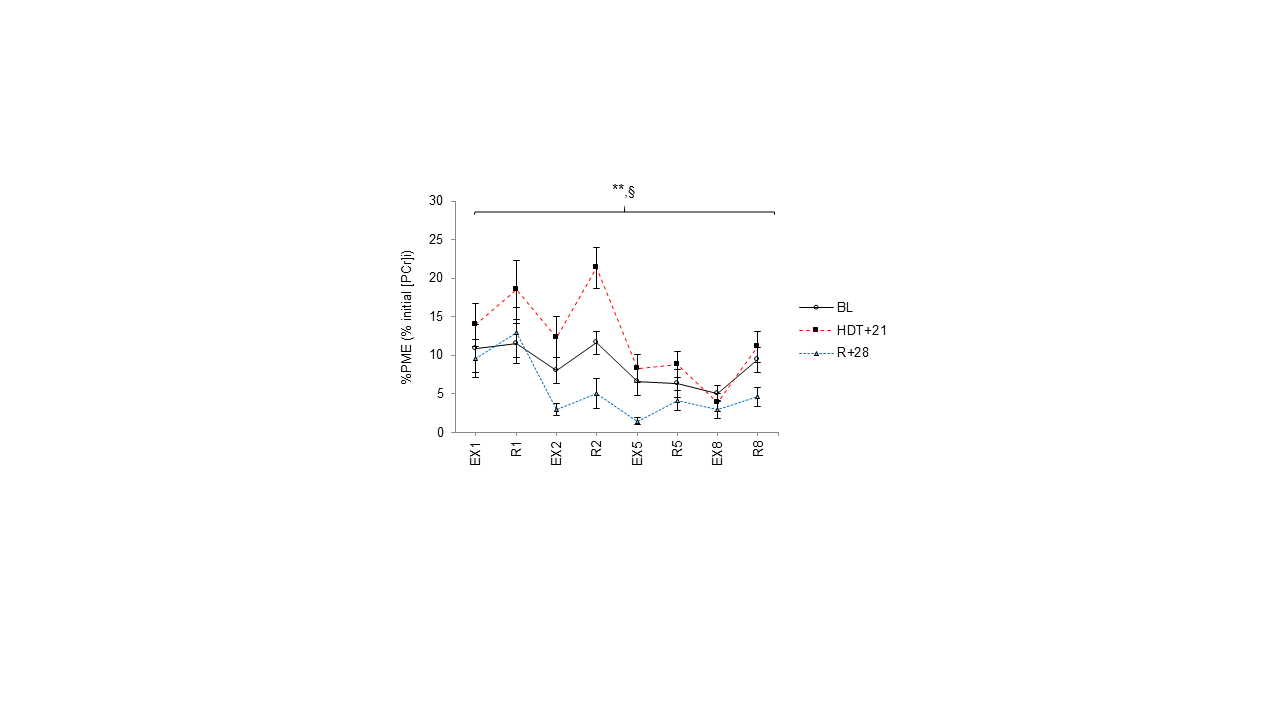

Supplement: Supplementary file 3 — Supplementary Fig. 3 The overall effect of bed rest on PME levels in the calf musculature during the fatigue test. **Condition effect at P<0.001. §Post bed rest data only recovery: Interval P<0.001; 2>5-8 at P≤0.015. BL: baseline; HDT+21: 21-day' bed rest (mean with/without WP+KHCO3); R+28: mean recovery period. The panel shows data at interval 1,2,5 and 8. Data are expressed as mean ± SD (TIF 60 kb) [file 421_2020_4333_MOESM3_ESM.tif]
